# iDNA-MT: Identification DNA Modification Sites in Multiple Species by Using Multi-Task Learning Based a Neural Network Tool

**DOI:** 10.3389/fgene.2021.663572

**Published:** 2021-03-31

**Authors:** Xiao Yang, Xiucai Ye, Xuehong Li, Lesong Wei

**Affiliations:** ^1^School of Software, Shandong University, Jinan, China; ^2^Department of Computer Science, University of Tsukuba, Tsukuba, Japan; ^3^Department of Rehabilitation, Heilongjiang Province Land Reclamation Headquarters General Hospital, Harbin, China

**Keywords:** multi-task learning, DNA modification, feature representation, deep learning, neural network

## Abstract

**Motivation:**

DNA N4-methylcytosine (4mC) and N6-methyladenine (6mA) are two important DNA modifications and play crucial roles in a variety of biological processes. Accurate identification of the modifications is essential to better understand their biological functions and mechanisms. However, existing methods to identify 4mA or 6mC sites are all single tasks, which demonstrates that they can identify only a certain modification in one species. Therefore, it is desirable to develop a novel computational method to identify the modification sites in multiple species simultaneously.

**Results:**

In this study, we proposed a computational method, called iDNA-MT, to identify 4mC sites and 6mA sites in multiple species, respectively. The proposed iDNA-MT mainly employed multi-task learning coupled with the bidirectional gated recurrent units (BGRU) to capture the sharing information among different species directly from DNA primary sequences. Experimental comparative results on two benchmark datasets, containing different species respectively, show that either for identifying 4mA or for 6mC site in multiple species, the proposed iDNA-MT outperforms other state-of-the-art single-task methods. The promising results have demonstrated that iDNA-MT has great potential to be a powerful and practically useful tool to accurately identify DNA modifications.

## Introduction

DNA modifications have been identified in multiple species. DNA modification plays an irreplaceable role in many basic biological functions (Fu and He, 2012; Shen and Zou, 2020). It refers to add methyl or hydroxymethyl groups to the nucleotides of DNA molecules. In particular, it is essential in the normal development of organisms such as aging, carcinogenesis, and X chromosome inactivation. Due to its importance, DNA methylation is one of the most widely studied epigenetic modifications (Bergman and Cedar, 2013; Smith and Meissner, 2013). Currently, four out of the DNA modifications, such as N4-methylcytosine (4mC), N6-methyladenine (6mA), 5-methylcytosine (5mC), and 5-hydroxymethylcytosine (5hmC), have been extensively studied (Cheng and Baldi, 2006; Guohua et al., 2017; He et al., 2019; Luo et al., 2020; Zuo et al., 2020c).

Schweizer (2008) proposed that 4mC has the effect of protecting the host DNA from degradation by restriction enzymes and belongs to restriction-modification (RM) systems. Timinskas et al. (1995) proposed 4mC can methylate the 4th amino group of cytosine in DNA under the catalysis of N-4 cytosine-specific DNA methyltransferase (DNMT). Iyer et al. (2011) proposed 4mC can distinguish the self and foreign DNA of prokaryotes and repair DNA replication errors. 5hmC arises from the oxidation of 5-methylcytosine (5mC) by Fe^2 +^ and 2-oxoglutarate-dependent 10–11 translocation (TET) family proteins (Hu et al., 2019). Thomson and Meehan (2016) proposed 5hmC can be used as an identifier of cell type or disease state. It is an intermediate product produced during the 5mC demethylation process. Szulwach et al. (2011) proposed 5hmC is critical in neurodevelopment and diseases (Tang et al., 2018; Zhang Y. et al., 2019). 6mA is a non-canonical DNA base modification present at low levels and maybe a carrier of heritable epigenetic information in eukaryotes (Greer et al., 2015; Mondo et al., 2017) and is found in the genomes of certain protists and fungi and might exist in other eukaryotes (Wion and Casadesús, 2006). The role of 6mA is very extensive. For example, it protects against restriction enzymes in bacteria (Heyn and Esteller, 2015) and unravels the DNA double helix structure during the cell cycle (Fang et al., 2012), which is catalyzed by two classes of DNA adenine methyltransferases (Wion and Casadesús, 2006; Zhang L. et al., 2019).

Numerous studies have shown that 5hmC, 6mA, and 4mC, and others are widely present in the genome, and significant progress has been made (Wu et al., 2016; Ao et al., 2019; Hu et al., 2019; Zhu et al., 2019; Zou et al., 2019; Cai et al., 2020; Fu et al., 2020; Hong et al., 2020). However, methylation-related technologies-the short-read sequencing and long-read have major disadvantages. For example, short-read technology can convert unmethylated cytosine to uracil. However, it has intrinsic disadvantages, such as low positioning efficiency and low accuracy. Long-read sequencing can be used to identify DNA modifications. There is a problem that it does not have a high signal-to-noise ratio for DNA modification. In nature, 5hmC, 6mA, and 4mC content are low, and the requirements for detection technology are relatively high. Therefore, we perform predictive calculations in advance, which can improve the efficiency of the experiment, to reduce the cost of the experiment, and provide guidance information for subsequent implementations.

Recently, there have been many machine learning methods to predict DNA methylation sites (Basith et al., 2019; Chen and Zou, 2019; Dou et al., 2020; Lv et al., 2020b). For instance, Ni et al. (2019) proposed DeepSignal, a deep learning approach to detect DNA methylation states from Nanopore sequencing reads. Besides, Liu et al. (2016) designed a two-way neural network with long short-term memory, called DeepMod. It can also identify DNA methylation sites in *E. coli* and *Homo sapiens*. Chen et al. (2019) developed a computational method called i6mA-Pred, to identify 6mA sites targeted to the rice genome, in which the optimal nucleotide chemical properties obtained by the using feature selection technique were used to encode the DNA sequences. Similarly, Yu and Dai (2019) created SNNRice6mA based on deep learning to identify 6mA in rice.

Kong and Zhang (2019) proposed a new machine learning-based method, namely i6mA-DNCP, which proved that there is also 6mA sites also in the rice genome. In i6mA-DNCP, dinucleotide composition and dinucleotide-based DNA properties were first employed to represent DNA sequences. Chen et al. (2017) developed iDNA4mC, the first webserver to identify 4mC sites, in which DNA sequences are encoded with both nucleotide chemical properties and nucleotide frequency. Later on, Wei et al. (2019b) developed a new predictor named 4mcPred-IFL to identify 4mC sites, in which they proposed an iterative feature representation algorithm that enables learning informative features from several sequential models in a supervised iterative mode. Basith et al. (2019) developed a novel computational predictor, called the Sequence-based DNA N6-methyladenine predictor (SDM6A), which is a two-layer ensemble approach for identifying 6mA sites in the rice genome. Manavalan et al. (2019a) designed the first method for identifying 4mC sites in the mouse genome, called 4mCpred-EL. Similarly, Hasan et al. (2020) invented a method to identify the 4mC sites, called i4mC-ROSE in the Fragaria vesca and Rosa genome. However, the training data of the above methods are all derived from specific species. And when extended to other species, it may produce a low true-positive rate with a high false-positive rate. Therefore, there is urgent to develop a generic DNA modification site predictor that can be used in different species. In other biological and medical fields, machine learning-based computational methods have been widely used, including microRNAs and cancer association prediction (Yuming et al., 2015; Jiang et al., 2018; Ding et al., 2020a; Wang et al., 2021), function prediction of proteins (Ding et al., 2019d, 2020b; Wang Y. et al., 2019; Wang H. et al., 2019; Tao et al., 2020; Zou et al., 2020b; Yang et al., 2021), drugs complex network analysis (Ding et al., 2017, 2019a,b,c, 2020c; Guo et al., 2020b) and dry weight assessment of hemodialysis patients (Guo et al., 2020a).

In this study, we developed a new deep learning-based multi-task method, called iDNA-MT, for identifying 4mC site and 6mA site in multiple species, respectively. This method combines both the bidirectional gated recurrent units (BGRU) and multi-task learning to learn sharing information hiding in different species for better characterizing a DNA sequence. Afterward, the sharing features are fed into the corresponding fully connected layers, specifically designed for a certain task, to identify the modification site. Several experiments were carried out to investigate the performance of the proposed iDNA-MT. Experimental results on two benchmark datasets showed that iDNA-MT achieved significantly better performance than state-of-the-art single-task methods for identifying 4mC site and 6mA site, respectively. In addition, our model can provide a powerful tool for identifying 4mC sites and 6mA sites in multiple species, respectively, and facilitate our knowledge of their biological functions.

## Materials and Methods

### Dataset

For a fair comparison, we employed the same benchmark datasets derived from Lv et al. (2020a). Four species of 4mC site data and four species of 6mA site data were selected. The 4mC site data contains four species (*C. equisetifolia, F. vesca, S. cerevisiae*, and *Tolypocladium*) that were collected from the MDR database (Liu et al., 2016) and MethSMRT database (Pohao et al., 2017). The 6mA site data for four species (*Tolypocladium, C. elegans, C. equisetifolia*, and *R. chinensis*) were extracted from the MethSMRT database (Pohao et al., 2017), MethSMRT database (Pohao et al., 2017), and MDR database (Liu et al., 2016). The benchmark data is divided into two parts. One part is used as a training dataset, and the other one is a testing dataset. The function of the training dataset is to train and evaluate the predictive model, while the purpose of the testing dataset is to test the performance of the model. The number of positive and negative samples is the same in the training dataset and testing dataset. A summary of the different species datasets used for benchmarking is displayed in Table 1.

**TABLE 1:** Summary of benchmark datasets used in this study.

Modifications	Species	Testing dataset	Training dataset
4mC	*C. equisetifolia*	365	365
	*F. vesca*	15,795	15,797
	*S. cerevisiae*	1,977	1,979
	*Tolypocladium*	15,325	15,327
6mA	*Tolypocladium*	3,377	3,379
	*C. elegans*	7,959	7,961
	*C. equisetifolia*	6,065	6,065
	*R. chinensis*	597	599

### Neural Network Architecture of the Proposed iDNA-MT

**FIGURE 1:**
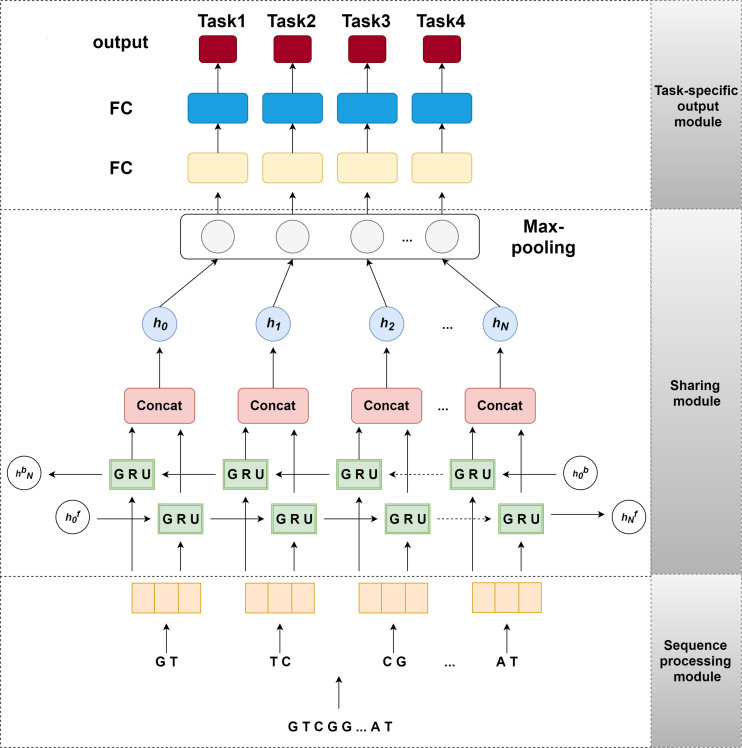
The structure of iDNA-MT. The sequence processing module uses 2-gram to split an original DNA sequence into overlapping subsequences and converts them into feature vectors by one-hot encoding. Next, the feature vectors of subsequences are sent into sharing module, containing a BGRU and a *max-pooling* layer, to capture the sharing information among different species. Finally, the output of sharing module is fed into the task-specific output module to predict the modification site (i.e., 4mA or 6mC) of a certain species.

In this section, we introduce the network architecture of our model iDNA-MT, as illustrated in Figure 1. This network architecture consists of three main components: (i) sequence processing module, (ii) sharing module, and (iii) task-specific output module. To make DNA sequences recognized easily by the neural network, the sequence processing module is designed to encode the original DNA sequences into matrices by one-hot encoding (Quang and Xie, 2016). Next, the encoded matrix is passed through a bidirectional GRU to extract different levels of dependency relationships between subsequences, and then a max-pooling layer is employed to automatically measure which feature plays a key role in NDA methylation site identification in each unit of the GRU. Finally, the features learned from the max-pooling layer are sent to the task-specific output module to identify 6mA sites in four species, respectively. The task-specific output module contains four parts and each part consists of fully connected layers that are designed in terms of the size of the training set of each species. The model is implemented using Pytorch. Below each module of our model is described in detail.

#### Sequence Processing Module

DNA modification identification is the task to separate the DNA sequences into related classes of DNA modifications, while text categorization is the problem of assigning text documents to predefined categories. To apply text categorization techniques to DNA sequences, we first employed n-gram nucleobases to define “words” in DNA sequences (Dong et al., 2006; Dao et al., 2020; Wang et al., 2020; Zhang et al., 2020). The n-grams are the set of all possible subsequences of nucleobases. Then, we split the DNA sequences into overlapping n-gram nucleobases. The number of possible it is 4^*n*^, since there are four types of nucleobases (Yang et al., 2020). In this study, to avoid low-frequency words in the encoding, the n-gram number n is set to 2. For example, we split a DNA sequence into overlapping 2-gram nucleobase sequences as follows: *GTTGT*…*CTT*→ *“GT,” “TT,” “TG,” “GT,”*…, *“CT,” “TT.”*

For a given DNA sequence P of length L, it can be expressed as follows:

(1) $$P=R_{1},R_{2},\ldots ,R_{L}$$

where *R*_*i*_ is the *i -*th word. These words are first randomly initialized embedded by one-hot embedding, which is referred to as “word embeddings.” Here, we defined the sequence of word embeddings as:

(2) $${\text{x}}_{1},{\text{x}}_{2},\ldots ,{\text{x}}_{L}$$

where *x*_*i*_ ∈ *ℝ*^*d*^ is the d-dimensional embedding of the *i -*th word. In the proposed method, such a sequence is fed into the bidirectional GRU to extract dependency information.

#### Sharing Module

##### Bidirectional Gated Recurrent Units

**FIGURE 2:**
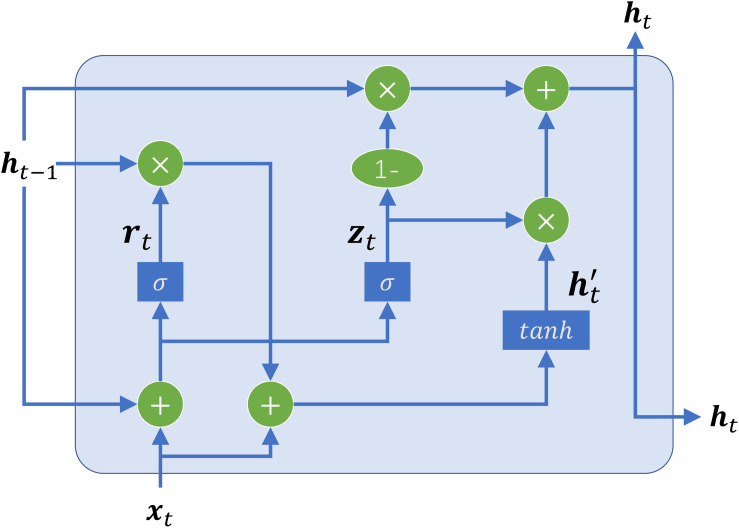
The structure of GRU cell. There are two gates, including a reset gate (denoted as ***r***_*t*_) and an update gate (denoted as ***z***_*t*_), to control the information flowing in and out of the cell. The reset gate control how much of the previous information to store. The update gate control how much of the past information needs to be passed along to the next time step. *x* is the embedding matrix of the input subsequence, *h* is the output of the GRU cell, and *t* denotes the *t* -th time step.

GRU is one of the widely used deep learning techniques, which is designed to specifically address the problems of learning long-distance correlations in a sequence (Cho et al., 2014). Bidirectional GRU is the most important part of the sharing module, which is employed to automatically extract long-terms and short-term dependency relationships in DNA sequences. The structure of the basic unit of GRU is shown in Figure 2. The unit receives two input vectors: the embedding vector of the subsequence and the hidden state of the previous time step. The special thing about them is that they can be trained to keep information from long ago. Based on the two inputs, two gates, namely, reset gate and update gate, coordinate with each other to capture short-term and long-term dependencies in sequences. The reset gate is used to control how much of the previous information to forget. Likewise, the update gate helps the model to determine how much of the past information, from previous time steps, needs to be passed along to the future.

For a given time step *t*, there are four components composite the GRU-based recurrent neural network: a reset gate ***r***_*t*_ with corresponding weight matrices ***W***_*r*_, ***U***_*r*_; an update gate ***z***_*t*_ with corresponding weight matrices ***W***_*z*_, ***U***_*z*_; a candidate hidden state ${\text{h}}_{t}^{\prime }$ with corresponding weight matrices ***W***,***U***; and a new hidden state ***h***_*t*_. The equations of GRU are the following:

(3) $${\text{r}}_{t}=\sigma \left({\text{W}}_{r}{\text{x}}_{t}+{\text{U}}_{r}{\text{h}}_{t-1}\right)$$

(4) $${\text{z}}_{t}=\sigma \left({\text{W}}_{z}{\text{x}}_{t}+{\text{U}}_{z}{\text{h}}_{t-1}\right)$$

(5) $${\text{h}}_{t}^{\prime }=tanh\left(\text{W}x_{t}+{\text{r}}_{t}⊙{\text{Uh}}_{t-1}\right)$$

(6) $${\text{h}}_{t}={\text{z}}_{t}⊙{\text{h}}_{t-1}+\left(1-{\text{z}}_{t}\right)⊙{\text{h}}_{t}^{\prime }$$

where ***x***_*t*_ denotes the input of the current time step, σ denotes the logistic sigmoid function to transform input values to the interval (0,1), ***h***_*t*−1_ denotes the output of the last time step, ⊙ denotes element-wise multiplication, and *tanh* is a non-linear activation function to ensure the values in the candidate hidden state remain in the interval (−1,1). Hence, the new hidden state ***h***_*t*_ holds information for the current step and previous steps and passes it down to the network.

However, a standard GRU network process a sequence in temporal order, resulting in that the outputs only contain the forward sequence information. To fully extract the information of a sequence, it is significant to capture not only the forward information but also the backward information at each time step. Therefore, we attempt to add another GRU network that captures the backward sequence information by processing a DNA sequence in the opposite temporal order. Combine it with the standard GRU network to form a bidirectional GRU, which can exploit information both from the past and the future.

To better capture the dependency information of subsequences with large time step distances, in this study, we combined the forward and backward hidden vectors generated by bidirectional GRU in each step. Therefore, the *i -*th subsequence can be expressed as the following vector:

(7) $${\text{h}}_{i}=\left({\text{h}}_{i}^{f},{\text{h}}_{i}^{b}\right)$$

where ***h*** is the hidden vector, ${\text{h}}_{i}^{f}$ and ${\text{h}}_{i}^{b}$ denote the hidden vectors generated by the forward GRU and the backward GRU, respectively.

##### Max-pooling Layer

The feature vector ***h*** of each subsequence, generated by bidirectional GRU, is fed into a *max-pooling* layer to capture the most significant feature in identifying the DNA modification to represent this subsequence. Then, all the most significant features of subsequences are concatenated into a vector to represent a DNA sequence, which is shown in the following equation:

(8) $$\text{y}=max_{i=1}^{n}{\text{h}}_{i}$$

where *i* is the *i* -th subsequence, *n* is the number of subsequences in a DNA sequence, and the ***y*** is regarded as the feature vector of a target sequence. The max-pooling layer attempts to find the most important dependencies in subsequences.

#### Task-Specific Output Module

This module consists of four sets of fully connected layers corresponding to each task, respectively. In each fully connected layer with a *relu* activation function, its output is calculated by the following equation:

(9) $${\text{f}}_{i}^{j}=relu\left({\text{W}}_{i}^{j}{\text{f}}_{i-1}^{j}+{\text{b}}_{i}^{j}\right)$$

where ${\text{f}}_{i-1}^{j}$ is the output of the previous layer of *j* -th task, ${\text{f}}_{i}^{j}$ is the current layer output of *j* -th task, ${\text{W}}_{i}^{j}$ is the weight matrix, and ${\text{b}}_{i}^{j}$ is the bias vector. In each layer, the “Batch Normalization” technique was used to improve generalization performance (Cheng and Baldi, 2006). Finally, a *softmax* layer is added on the top of final output ***f***^*j*^ to perform the final prediction. Note that the parameters of different set of the fully connected layer are designed differently in terms of the amount of data of the corresponding task.

### Training

The task-specific features ***y***, generated by the sharing module, are ultimately sent into one set of fully connected layers in terms of it belonging to which task. For classification tasks, we used binary cross-entropy loss function as the objective:

(10) $$l=\frac{1}{N}\sum_{i}-\left[y_{i}log\left(p_{i}\right)+\left(1-y_{i}\right)log\left(1-p_{i}\right)\right]$$

where *N* denotes the number of training samples, *y*_*i*_ denotes the label (i.e., 1 or 0) of sample *i, p*_*i*_ denotes the probability that sample *i* is predicted to be positive. Our global loss function is the linear combination of loss function for all tasks:

(11) $$l_{all}=\sum_{k=1}^{k}\alpha_{k}l_{k}$$

where α_*k*_ is the weight for task *k*.

It is worth noting that the samples for training each task can come from completely different datasets. Following the study (Liu et al., 2016), the training is carried out in a stochastic manner by looping over the tasks:

1. Select a task randomly.
2. Select a training sample from this task randomly.
3. Update the parameters for this task by taking a gradient step in terms of this sample.
4. Go to 1.

### Evaluation Metrics

To evaluate the performance of our model, four commonly used metrics are employed to evaluate the performance of the model (Zou et al., 2016; Jin et al., 2019, 2020; Manayalan et al., 2019; Manavalan et al., 2019b; Hong et al., 2020; Lv et al., 2020b; Qiang et al., 2020; Su et al., 2020a,b,c, 2019a,b; Wei et al., 2020, 2014, 2019a, 2018a,b; Zhao et al., 2020; Zou et al., 2020a), including sensitivity (SN), specificity (SP), overall accuracy (ACC), and Matthew’s correlation coefficient (MCC), respectively. They are formulated as:

(12) $$SN=\frac{TP}{TP+FN}$$

(13) $$SP=\frac{TN}{TN+FP}$$

(14) $$ACC=\frac{TP+TN}{TP+FN+TN+FP}$$

(15) $$MCC=\frac{\left(TP\times TN\right)-\left(FP\times FN\right)}{\sqrt{\left(TP+FN\right)\times \left(TP+FP\right)\times \left(TN+FN\right)\times \left(TN+FP\right)}}$$

where TP, TN, FP, and FN represent the numbers of true positives, true negatives, false positives, and false negatives, respectively. SN and SP are used to evaluate positive and negative predictive ability. MCC and ACC were used to evaluate the overall prediction performance. Besides, the ROC curve (receiver operating characteristic curve) can be used to visualize the performance of the classifier. In addition, we calculate the area under the ROC curve (AUC) to evaluate the prediction performance of the model. The range of AUC is 0.5–1. The higher the AUC score, the better the prediction performance of the model.

## Results and Discussion

### Performance Comparison With the State-of-the-Art Methods

To evaluate the performance of our model iDNA-MT for identifying 4mC and 6mA site in multiple species, we compared it with seven state-of-the-art models based on random forest (RF), which were all single-task learning methods and used different feature descriptors to identify 4mC and 6mA site in each species, respectively, including K-tuple nucleotide frequency component (KNFC), nucleotide chemical property and nucleotide frequency (NCPNF), and mono-nucleotide binary encoding (MNBE), and their four combinations (Lv et al., 2020a).

**FIGURE 3:**
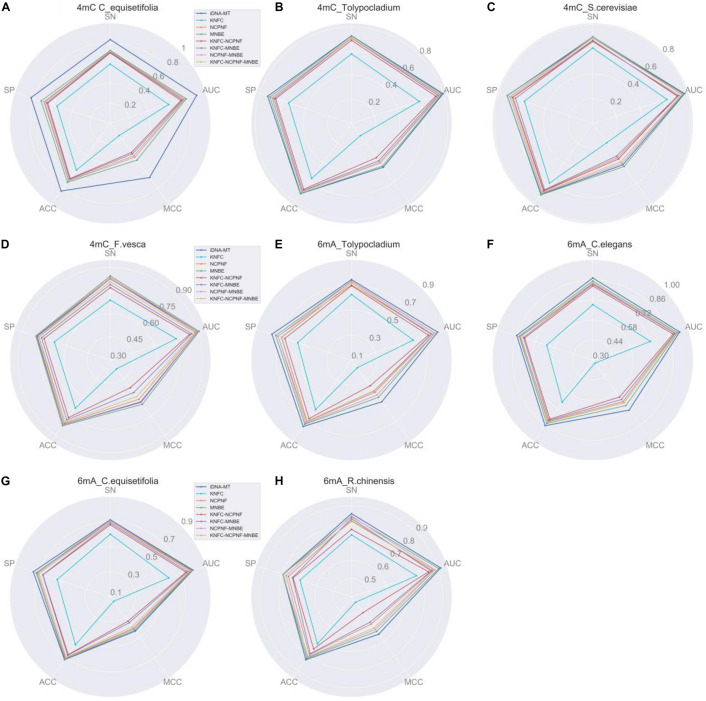
Performance evaluation of iDNA-MT and seven state-of-the-art methods for identifying 4mC site and 6mA site. **(A–D)** represents the performance of identifying 4mC site. **(E,F)** represents the performance of identifying 6mA site. **(A–D)** represent the performance of different methods for identifying 4mC site. **(E–H)** represent the performance of different methods for identifying 6mA site.

The experimental results of different methods are listed in Figure 3. From Figure 3, we can observe that for 4mC site identification, our proposed iDNA-MT significantly outperforms all the other competing methods in three species (*C. equisetifolia*, *Tolypocladium*, and *S. cerevisiae*) in terms of five metrics (SN, SP, ACC, MCC, and AUC), while the model using MNBE achieves the best performance amongst all methods. For 6mA site identification, iDNA-MT exhibits better performance than any other RF-based models in each species. These results indicate that using both BGRU and multi-task learning can extract more effective and discriminative features to represent DNA sequences for identifying 4mA site and 6mC site and be generalized well on different species. There are two main reasons for the outstanding performance of our model. First, compared with the RF-based methods that use handcrafted features to train models, which need prior knowledge, iDNA-MT can automatically capture effective features by data driving. Second, the proposed iDNA-MT employs the BGRU to learn long-distance dependency information of DNA subsequences, and then introduce the multi-task learning technique to capture the shared information hidden in data from different species to improve the performance of each task, to improve the accuracy for identifying 4mC and 6mA site in multiply species, respectively. Therefore, iDNA-MT can achieve better performance than other state-of-the-art single-task learning methods. Note that the detailed comparison results of iDNA-MT and seven state-of-the-art methods can be found in Supplementary Table S1.

### Effectiveness of Multi-Task Learning

To evaluate whether or not introducing the multi-task learning technique can capture more discriminative features to improve the performance of DNA modification site prediction in multiple species, we compared the model considering the multi-task learning, namely iDNA-MT, with the model not considering the multi-task learning for prediction. The comparative results for 4mC site and 6mA site are illustrated in Tables 2, 3, respectively. In Tables 2, 3, we show better results in bold.

**TABLE 2:** Comparison results of the model using the multi-task learning and the model not using the multi-task learning for identifying 4mC site.

Modification type	Genome		SN (%)	SP (%)	ACC (%)	MCC	AUC
4mC	*C. equisetifolia*	Single	77.05	**85.79**	81.42	0.6308	0.8551
		Multi	**83.61**	83.06	**83.33**	**0.6667**	**0.9049**
	*Tolypocladium*	Single	69.94	72.61	71.28	0.4357	0.7837
		Multi	**72.72**	**73.12**	**72.09**	**0.4489**	**0.7989**
	*S. cerevisiae*	Single	66.23	**72.91**	69.57	0.3922	0.7520
		Multi	**69.32**	72.88	**71.09**	**0.4139**	**0.7765**
	*F. vesca*	Single	**83.48**	**82.06**	**82.77**	**0.6544**	**0.9047**
		Multi	82.67	79.86	81.79	0.6354	0.8966

**TABLE 3:** Comparison results of the model using the multi-task learning and the model not using the multi-task learning for identifying 6mA site.

Modification type	Genome		SN (%)	SP (%)	ACC (%)	MC C	AUC
6mA	*Tolypocladium*	Single	73.96	74.25	74.91	0.5001	0.8170
		Multi	**74.25**	**76.73**	**75.49**	**0.5110**	**0.8222**
	*C. elegans*	Single	**87.51**	85.55	86.53	0.7308	0.9334
		Multi	87.39	**85.73**	**86.56**	**0.7313**	**0.9374**
	*C. equisetifolia*	Single	69.47	71.12	70.29	0.4059	0.7696
		Multi	**71.45**	**74.55**	**72.01**	**0.4385**	**0.7923**
	*R. chinensis*	Single	78.93	72.24	75.85	0.5129	0.8334
		Multi	**85.62**	**79.62**	**82.61**	**0.6534**	**0.9134**

As shown in Table 2 for 4mC site prediction, we can see that training with the multi-task learning, the model achieves higher performance in three species, including *C. equisetifolia, Tolypocladium*, and *S. cerevisiae*, with only one exception in *F. vesca*. Specifically, the model using the multi-task learning achieves an ACC of 83.33%, an MCC of 0.6667, and an AUC of 0.9049 for species *C. equisetifolia*, yielding a relative improvement of 2.3%, 5.7%, and 5.8%, respectively, achieves an ACC of 72.09%, an MCC of 0.4489 and an AUC of 0.7989 for species *Tolypocladium*, yielding a relative improvement of 1.1%, 3.0%, and 1.9%, respectively, and achieves an ACC of 71.09%, an MCC of 0.4139 and an AUC of 0.7765 for species *S. cerevisiae*, yielding a relative improvement of 2.2%, 5.5%, and 3.3%, respectively. For species *F. vesca*, the model using multi-task learning is slightly worse than the model not using multi-task learning, which achieves 82.67%, 79.86%, 81.79%, 0.6354, and 0.8966 in terms of SN, SP, ACC, MCC, and AUC. From Table 3, we can see that for all four species (*Tolypocladium, C. elegans, C. equisetifolia*, and *R. chinensis*), the model using multi-task learning all significantly outperforms the model not using multi-task learning for identification 6mA site in terms of SN, SP, ACC, MCC, and AUC. The most significant improvement is observed in species *R. chinensis*, in which the model using multi-task learning improves the SN from 78.93% to 85.62%, the SP from 72.24% to 79.62%, the ACC from 75.85% to 82.61%, the MCC from 0.5129 to 0.6534 and the AUC from 0.8334 to 0.9134.

These results discussed above demonstrate that by introducing the multi-task learning, the model can achieve outstanding performance for 4mC site and 6mA site prediction in multiply species, respectively. The reason may be that multi-task learning aims to learn shared representations from multiple related tasks, which are used to share and supplement the information learned from different tasks to improve the performance of multiple related learning tasks. Therefore, there is not surprising that the model using multi-task learning significantly outperforms the model not using multi-task learning.

### Performance of the Neural Network Architecture in Sharing Module

The sharing module of iDNA-MT mainly employed BGRU to exploit the potential information both from forward and backward and then used the *max-pooling* layer to extract the most significant features in subsequences, which play key roles in DNA modification identification. To evaluate the efficiency and superiority of the neural network architecture in sharing module, we replaced it with other three typical text classification methods, respectively, including:

1. TextRNN (Liu et al., 2016): It uses the long short-term memory network (LSTM) to capture long-term semantic dependencies in a sentence.
2. Att-BLSTM (Zhou et al., 2016): It utilizes both neural attention mechanism and bidirectional long short-term memory networks (BLSTM) to capture the most important semantic information in a sentence.
3. Transformer (Vaswani et al., 2017): It is a novel neural network architecture based on a self-attention mechanism.

**FIGURE 4:**
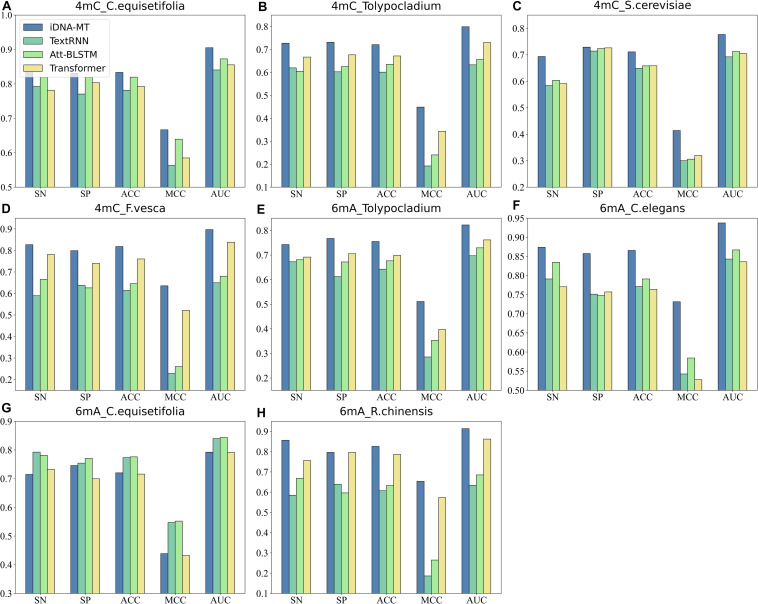
Performance evaluation of iDNA-MT and other methods using different typical text classification methods in sharing module **(A–D)** represent the performance of iDNA-MT and other methods for identifying 4mC site. **(E–H)** represent the performance of iDNA-MT and other methods for identifying 6mA site.

Figure 4 shows the comparison results of the proposed iDNA-MT and the other methods using different typical text classification methods in sharing modules on two different modification sites in terms of five metrics (SN, SP, ACC, MCC, and AUC). As shown in Figure 4, we can see that for 4mC site, the performance of iDNA-MT is significantly better than the other methods using different typical text classification methods in sharing module in every species. For 6mA site, although the performance of iDNA-MT is lower than other methods in species *C. equisetifolia*, the performance of iDNA-MT significantly outperforms other methods in the rest species. Therefore, iDNA-MT is superior to other methods in identifying 4mC sites and 6mA sites in multiple species, respectively. The proposed iDNA-MT used BGRU to capture the dependency information of subsequences from the past and the future and added a *max-pooling* layer to extract the most important information hiding in every subsequence, which avoids irrelevant information from interfering with identifying results. Therefore, there is no surprise that iDNA-MT achieves the best performance when combing BGRU and a *max-pooling* layer.

## Conclusion

Although 4mA and 6mC are two important genetic modifications and play crucial roles in regulating a series of biological processes, their biological functions are still unclear. Therefore, the accurate identification of them is pivotal to understand specific biological functions. In this study, we proposed a multi-task learning predictor namely iDNA-MT for identifying 4mA site and 6mC site in multiple species, respectively, which can automatically extract the discriminative features for different tasks. To better represent the DNA sequences of different species, we constructed a sharing module, containing a BGRU and a *max-pooling* layer, to capture sharing information among different species. To evaluate the efficiency of our multi-task model, we compared it with the state-of-the-art single-task models on benchmark datasets of two different DNA modifications. Experimental results have shown that the proposed iDNA-MT achieved the top performance comparing with existing single-task models on two benchmark datasets, indicating that multi-task learning can improve the performance of multiple related tasks by leveraging useful information among them. In future work, we would like to investigate other sharing mechanisms to further improve the prediction of different DNA modifications in multiple species and apply it to other fields (Wei et al., 2017a,b,c, 2018c, 2019c,d; Zou et al., 2019).

## Data Availability Statement

All datasets generated for this study are included in the article/Supplementary Material, further inquiries can be directed to the corresponding author/s.

## Author Contributions

XY and LW surveyed the algorithms and implementations, preprocessed the datasets, and performed all the analyses. XCY and XL designed the benchmarking test. All the authors have written, read, and approved the manuscript.

## Conflict of Interest

The authors declare that the research was conducted in the absence of any commercial or financial relationships that could be construed as a potential conflict of interest.
