# Supplementary material for: iDNA-MT: Identification DNA Modification Sites in Multiple Species by Using Multi-Task Learning Based a Neural Network Tool
Source: Front Genet. 2021 Mar 31;12:663572. doi: 10.3389/fgene.2021.663572 (PMC8044371; doi:10.3389/fgene.2021.663572)
Supplement: Supplementary file 1 [file Table_1.docx]

**Supporting Information**

Table S1. Performance evaluation of iDNA-MT and seven state-of-the-art methods for identifying 4mC site and 6mA site.

| Modification  type | Genome | Performance | KNFC | NCPNF | MNBE | KNFC-  NCPNF | KNFC-  MNBE | NCPNF-  MNBE | KNFC-NCPNF-  MNBE | iDNA-MT |
| --- | --- | --- | --- | --- | --- | --- | --- | --- | --- | --- |
| 4mC | C.equisetifolia | SN(%) | 59.20 | 70.94 | 72.75 | 70.28 | 70.90 | 72.08 | 71.59 | **83.61** |
|  |  | SP(%) | 55.83 | 68.93 | 72.48 | 65.46 | 66.58 | 69.55 | 68.48 | **83.06** |
|  |  | ACC(%) | 57.51 | 69.94 | 72.62 | 67.87 | 68.74 | 70.81 | 70.04 | **83.33** |
|  |  | 𝑀𝐶C | 0.150 | 0.399 | 0.452 | 0.358 | 0.375 | 0.416 | 0.401 | **0.6667** |
|  |  | AUC | 0.612 | 0.768 | 0.796 | 0.739 | 0.755 | 0.781 | 0.771 | **0.9049** |
|  | Tolypocladium | SN(%) | 57.72 | 70.56 | 72.56 | 68.95 | 70.848 | 71.26 | 70.85 | **72.72** |
|  |  | SP(%) | 54.83 | 69.25 | 71.14 | 66.18 | 67.46 | 69.39 | 68.85 | **73.12** |
|  |  | ACC(%) | 56.28 | 69.90 | 71.85 | 67.56 | 69.15 | 70.32 | 69.85 | **72.09** |
|  |  | 𝑀𝐶C | 0.126 | 0.398 | 0.437 | 0.351 | 0.383 | 0.407 | 0.397 | **0.4489** |
|  |  | AUC | 0.592 | 0.768 | 0.788 | 0.734 | 0.753 | 0.776 | 0.766 | **0.7989** |
|  | S.cerevisiae | SN(%) | 61.11 | 66.16 | 70.30 | 67.27 | 66.97 | 69.39 | 69.09 | 69.32 |
|  |  | SP(%) | 58.18 | 67.98 | 72.83 | 68.18 | 65.76 | 70.61 | 69.29 | **72.88** |
|  |  | ACC(%) | 59.65 | 67.07 | **71.57** | 67.73 | 66.36 | 70.00 | 69.19 | 71.09 |
|  |  | 𝑀𝐶C | 0.193 | 0.341 | **0.431** | 0.355 | 0.327 | 0.400 | 0.384 | 0.4139 |
|  |  | AUC | 0.631 | 0.735 | **0.783** | 0.718 | 0.723 | 0.764 | 0.758 | 0.7765 |
|  | F.vesca | SN(%) | 68.92 | 82.71 | **84.58** | 76.97 | 79.01 | 83.57 | 80.71 | 82.67 |
|  |  | SP(%) | 68.05 | 78.66 | **80.78** | 75.10 | 76.83 | 80.40 | 78.47 | 79.86 |
|  |  | ACC(%) | 68.48 | 80.68 | **82.68** | 76.03 | 77.92 | 81.99 | 79.59 | 81.79 |
|  |  | 𝑀𝐶C | 79.59 | 0.614 | **0.654** | 0.521 | 0.559 | 0.640 | 0.592 | 0.6354 |
|  |  | AUC | 0.749 | 0.884 | **0.905** | 0.839 | 0.854 | 0.898 | 0.875 | 0.8966 |
| 6mA | Tolypocladium | SN(%) | 62.37 | 70.06 | 73.02 | 69.35 | 71.78 | 72.90 | 71.72 | **74.25** |
|  |  | SP(%) | 55.15 | 71.60 | 73.55 | 65.92 | 68.58 | 71.54 | 68.88 | **76.73** |
|  |  | ACC(%) | 58.76 | 70.83 | 73.28 | 67.63 | 70.18 | 72.22 | 70.30 | **75.49** |
|  |  | 𝑀𝐶C | 0.176 | 0.417 | 0.466 | 0.353 | 0.404 | 0.444 | 0.406 | **0.5110** |
|  |  | AUC | 0.617 | 0.777 | 0.798 | 0.744 | 0.767 | 0.797 | 0.778 | **0.8222** |
|  | C.elegans | SN(%) | 68.78 | 82.92 | 85.28 | 81.79 | 83.14 | 83.55 | 84.15 | **87.39** |
|  |  | SP(%) | 63.78 | 82.87 | 83.95 | 79.98 | 80.86 | 83.35 | 82.69 | **85.73** |
|  |  | ACC(%) | 66.28 | 82.89 | 84.61 | 80.88 | 82.00 | 83.45 | 83.42 | **86.56** |
|  |  | 𝑀𝐶C | 0.326 | 0.658 | 0.692 | 0.618 | 0.640 | 0.669 | 0.668 | **0.7313** |
|  |  | AUC | 0.723 | 0.904 | 0.922 | 0.888 | 0.902 | 0.913 | 0.913 | **0.9374** |
|  | C.equisetifolia | SN(%) | 59.97 | 69.83 | 70.79 | 67.49 | 68.88 | 70.89 | 69.83 | **71.45** |
|  |  | SP(%) | 54.50 | 71.25 | 72.07 | 66.47 | 66.67 | 70.89 | 69.73 | **74.55** |
|  |  | ACC(%) | 57.24 | 70.54 | 71.43 | 66.98 | 67.77 | 70.89 | 69.78 | **72.01** |
|  |  | 𝑀𝐶C | 0.145 | 0.411 | 0.429 | 0.340 | 0.356 | 0.418 | 0.396 | **0.4385** |
|  |  | AUC | 0.591 | 0.775 | 0.786 | 0.734 | 0.748 | 0.779 | 0.763 | **0.7923** |
|  | R.chinensis | SN(%) | 74.00 | 82.00 | 84.00 | 77.00 | 83.00 | 84.33 | 81.33 | **85.62** |
|  |  | SP(%) | 69.67 | 76.33 | 79.33 | 73.67 | 74.00 | 77.67 | 79.67 | **79.62** |
|  |  | ACC(%) | 71.83 | 79.17 | 81.67 | 75.33 | 78.50 | 81.00 | 80.50 | **82.61** |
|  |  | 𝑀𝐶C | 0.437 | 0.584 | 0.634 | 0.507 | 0.572 | 0.621 | 0.610 | **0.6534** |
|  |  | AUC | 0.774 | 0.867 | 0.902 | 0.844 | 0.859 | 0.880 | 0.877 | **0.9134** |
